# Should the nail plate be replaced or discarded after nail bed repair in children? Nail bed INJury Analysis (NINJA) randomised controlled trial: a health economic and statistical analysis plan

**DOI:** 10.1186/s13063-020-04724-1

**Published:** 2020-10-07

**Authors:** Jamie R. Stokes, May Ee Png, Abhilash Jain, Aina V. H. Greig, Beverly A. Shirkey, Melina Dritsaki, Jonathan A. Cook

**Affiliations:** 1 Oxford Clinical Trials Research Unit, Botnar Research Centre, Centre for Statistics in Medicine, Nuffield Department of Orthopaedics, Rheumatology, and Musculoskeletal Sciences, University of Oxford, Windmill Road, Oxford, OX3 7LD UK; 2 Nuffield Department of Primary Care Health Sciences, University of Oxford, Oxford, OX2 6GG UK; 3 Department of Plastic and Reconstructive Surgery, Imperial College Healthcare NHS Trust, London, UK; 4 Department of Plastic and Reconstructive Surgery, Guy’s and St Thomas’ NHS Foundation Trust, London, UK; 5 School of Social and Community Medicine, University of Bristol, Canynge Hall, Bristol, UK; 6 Surgical Intervention Trials Unit, Botnar Research Centre, Nuffield Department of Orthopaedics, Rheumatology, and Musculoskeletal Sciences, University of Oxford, Windmill Road, Oxford, OX3 7LD UK

**Keywords:** Nail, Paediatrics, Trauma, Replace, Discard, Statistic, Economic, Analysis plan, Trial

## Abstract

**Background:**

Nail bed trauma is one of the most common surgically treated paediatric hand injuries in the UK. Despite surgeons generally expressing a preference to replace the nail plate after repairing the nail bed, there is limited evidence to support this practice. We describe a statistical and health economic analysis plan (SHEAP) for the Nail bed INJury Analysis (NINJA) randomised controlled trial.

**Methods/design:**

NINJA is a multicentre, pragmatic, superiority, parallel group randomised controlled trial of the treatment of nail bed injury in participants 16 years old or younger. The study aims to evaluate the efficacy and cost-effectiveness of replacing the nail plate compared to discarding it following the repair of a nail bed injury. Surgical site infection at 7–10 days post-randomisation and cosmetic appearance of the nail are the co-primary outcomes for NINJA. Surgical site infection at 7–10 days post-randomisation will be evaluated using a logistic regression model adjusting for site as the sole stratification factor and allowing for intra-site correlation. Cosmetic appearance will be assessed via the newly developed Oxford Finger Nail Appearance Score and will be evaluated by use of a Mann-Whitney *U* test. An ordinal logistic regression model will also be used to assess the Oxford Finger Nail Appearance Score, adjusting for site and allowing for intra-site correlation. Secondary outcomes are measured at 7–10 days and 4 months and include the EQ-5D-Y questionnaire, pain at first dressing change, cost-effectiveness, late surgical site infection, and participant/parent satisfaction with nail healing. Missing primary outcome data will be summarised by treatment arm and investigated through a sensitivity analysis. Full details of the planned methods of analysis and descriptive statistics are described in this paper. The NINJA study protocol has been published previously.

**Discussion:**

The planned analysis strategy for the NINJA trial has been set out here to reduce the risk of reporting bias and data-driven analysis. Any deviations from the SHEAP described in this paper will be detailed and justified fully in the final report of the trial.

**Trial registration:**

ISRCTN, ISRCTN44551796. Registered on 23 April 2018.

## Background

Nail bed injuries are one of the most common forms of paediatric hand trauma, with approximately 10,000 cases treated annually in the UK [1]. Despite the frequency of these injuries, controversy remains around the appropriate treatment of nail bed injuries [2, 3]. Surgical treatment of these injuries involves removing the nail plate and repairing the underlying nail bed laceration with sutures. Once the nail bed has been repaired, 96% of surgeons in the UK will replace the nail plate [1]. The rationale behind replacing the nail is that it protects the nail bed repair site and acts as a splint by holding open the nail fold, which prevents scar adhesions (synechiae) between the nail fold and nail bed.

A recent study [4] has suggested that replacement of the nail plate is associated with an increase in complications, in particular the development of post-operative infections. The reason behind this apparent increase in infective and wound healing complications is thought to be that the nail plate acts as a foreign body, trapping bacteria and making infection more likely. If this is the case, discarding the nail plate could result in a reduction in incidence of infection and would also reduce the burden on the NHS in terms of follow-up visits, antibiotic courses, and hospital readmission [4]. No randomised controlled trials have yet been conducted to compare different methods of surgical management following nail bed repairs. The British Society for Surgery of the Hand (BSSH) and the British Association of Plastic, Reconstructive and Aesthetic Surgeons (BAPRAS) supported a national survey of clinicians and parents/patients and audit [1]. This led to the NINJA-P pilot study [5] being carried out, which formed the basis for the full NINJA trial.

The aim of the Nail bed INJury Analysis (NINJA) study is to conduct a randomised controlled trial to compare replacing the nail plate and discarding the nail plate following the repair of a nail bed injury in children aged 16 or under, in terms of both post-operative surgical site infection and the cosmetic appearance of the nail.

This paper describes the methods set out in the trial’s statistical and health economic analysis plan (SHEAP) and has been prepared in accordance with the published guidelines on the content of statistical analysis plans [6]. The aim of this paper is to provide transparency on the planned analysis strategies assessing both the clinical outcomes and cost-effectiveness of replacing the nail versus discarding the nail, in order to reduce the likelihood of reporting bias and data-driven results. The NINJA trial is registered in the International Standard Randomised Controlled Trials database, registration number ISRCTN44551796.

## Methods and design

### Trial design

NINJA is a multicentre, two-arm, parallel group, superiority, pragmatic randomised controlled trial. A total of 416 patients will be recruited from 20 centres across the UK over a period of 20 months. Participants will be randomised to either have the fingernail replaced or discarded following repair of the nail bed injury. Patients eligible for the trial will be randomised to the treatments in a 1:1 ratio, stratified by study site using blocks of size 2 and 4 in a 1:1 ratio. Randomisation will be performed using a secure online randomisation system when the participant is in the anaesthetic room prior to surgery, or as close to the time of surgery as possible. Participants will be followed up by their local clinics at the first routine clinic appointment at around 7–10 days post-surgery, and additionally via postal questionnaire at 4–12 months. The trial will run for 3 years. Details of the trial design and procedures have been published in full in the NINJA study protocol [7]. This study has been conducted as part of the portfolio of trials in the registered UKCRC Oxford Clinical Trials Research Unit (OCTRU) at the University of Oxford. It has followed their Standard Operating Procedures ensuring compliance with the principles of Good Clinical Practice and the Declaration of Helsinki and any applicable regulatory requirements.

### Current status of trial

Recruitment for the NINJA trial began in July 2018 and concluded in July 2019, recruiting 451 patients. Follow-up was completed for all patients in May 2020. The SHEAP was finalised and submitted for publication prior to the beginning of the final analysis.

### Objectives

The primary objective of the NINJA trial is to assess the effects of replacing or discarding the fingernail following a nail bed repair by comparing both the risk of surgical site infection and cosmetic appearance. Secondary objectives of the trial include assessing differences in participants’ health-related quality of life, pain at first dressing change, late infection, and satisfaction with nail healing. A within-trial economic analysis to assess the cost-effectiveness of replacing versus discarding the nail plate is also a secondary objective of the study.

### Outcomes

#### Primary outcomes

The co-primary outcome measures for this study are confirmed incidence of surgical site infection and a modified Zook score assessment of the cosmetic appearance of the nail after follow-up.

##### Surgical site infection

Data on incidence of surgical site infection at 7–10 days post-surgery will be collected. The process for the collection of early infection data, which has been designed to maximise data retention, is as defined below.

- The 7–10-day clinic form will be the main source of infection data for the primary outcome. The relevant timeframe for the primary infection outcome will be those recorded or treated within 15 days post-randomisation.
- In the case of a missing 7–10-day form, the process will be as follows:
  - ➢ Query with site to check if 7–10-day form can be retrieved for this participant.
  - ➢ If available, use a confirmation of additional treatment (CAT) form to supplement this missing information on infection, if the date of treatment on the form is within 15 days post-randomisation.
  - ➢ If neither the 7–10-day nor the CAT forms are available, if an infection is reported by the participant on the 4-month follow-up form, go back to site for clinical notes regarding this. If the site confirms that an infection reported on the 4-month form occurred within 15 days post-randomisation, this counts a valid infection for the primary outcome.
  - ➢ If neither the 7–10-day nor the CAT forms are available, but the participant reports no infection on their 4-month follow-up form, “no problem” will be imputed back into the primary outcome.
- If a participant’s 7–10-day form reported no infection, but a CAT form/site following a 4-month form reported a confirmed infection within the 15-day post-randomisation window, this information would supersede the 7–10-day form’s “no” for occurrence of an infection.
- If none of the above is possible/available after query, the participant has missing outcome data.

##### Oxford Finger Nail Appearance Score

The cosmetic appearance of the injured nail will be assessed by independent reviewers at 4 months post-randomisation. The reviewers will consist of trainee plastic surgeons, specialist registrars, and hand therapists. Reviewers will assess photographs taken at 4 months post-randomisation showing both the injured finger and the contralateral finger and complete the Oxford Finger Nail Appearance Score (based on the Zook classification scale [8]) giving a measure of the overall appearance of the nail. Reviewers will be informed which of the fingers is the injured finger, but will be blinded to the intervention received.

The Oxford Finger Nail Appearance Score is made up of five scoring components. These components assess the nail shape, the eponychium, the adherence of the nail plate to the nail bed, the nail surface, and the presence of a split in the nail. The score has been modified for the purpose of this study to contain only two scoring dimensions for each component. Nail shape, eponychium, and surface will be scored as either identical or not identical to the contralateral finger; nail adherence will be scored as either complete or incomplete; and nail split will be scored as either present or absent. If reviewers believe the nail differed in shape, eponychium, or surface in comparison to the contralateral nail, they will also be asked specifically why they thought this (e.g. “The injured nail appears narrower to the contralateral nail”). Reviewers will also be asked if, in their opinion, enough of the nail has grown back in order to make a valid assessment of the nail.

Each component will be scored as a 1 if it is deemed to be the same as opposite finger or not having the defect, and zero if the fingernail is deemed to be worse than the opposite finger or if the defect is present. The component scores will then be totalled, and each nail will receive a score between 0 (worst possible cosmetic score) and 5 (best possible cosmetic score). As five assessors will be scoring each photograph, the median of these values will be taken as the outcome for each nail.

#### Secondary outcomes

##### Health-related quality of life

The EQ-5D-Y is a validated, child-friendly, health-related quality of life (HRQoL) questionnaire consisting of five domains related to daily activities (mobility, looking after myself, usual activity, pain or discomfort, and feeling worried, sad, or unhappy) with 3-level answer possibilities (no problems, some problems, and a lot of problems). This will be completed by the patient if they are 7 years old and above or via parent/guardian proxy if the patient is between 2 and 6 years old, at baseline, 7–10 days, and 4–12 months post-randomisation.

In line with the age-centric languages of the NINJA trial’s patient information sheets, EQ-5D-Y (proxy) will be used for children aged 2–6 years while EQ-5D-Y will be used for children aged 7 years and above. For participants aged 2–4 years, the Paediatric Quality of Life Inventory (PedsQL) Generic Scale, a non-preference-based HRQoL instrument that has been designed to provide a modular approach to measure HRQoL among children and adolescents aged 2–18 years [9], was used during the early stages of the trial but was replaced by EQ-5D-Y (proxy). This is because the mapping algorithm used to estimate the health utilities from PedsQL is age group specific and there are currently no mapping algorithms available for children aged seven and under. Although EQ-5D-Y has not been validated for children under four and the proxy version is recommended only for ages 4–6 [10], given that there are currently no guidelines to collect HRQoL data from children under four and no valid algorithm to map PedsQL to health utilities, EQ-5D-Y (proxy) will be used for children aged 2–4 years. There will not be any measurement of HRQoL for children below 2 years old after reviewing the literature [11] and consulting the experts (EuroQoL advisors and experts in paediatrics health) as there is currently no validated measure of HRQoL for these ages.

##### Pain at dressing change

The level of pain experienced by the child at their first dressing change (completed at the 7–10-day follow-up clinic) will be assessed by use of a 3-point Likert scale. This will be completed by the patient, or a parent/guardian proxy if the child is not able to complete the score.

##### Cost-effectiveness

A within-trial cost-effectiveness analysis (CEA) will be undertaken to assess the cost-effectiveness of replacing versus discarding the nail. The base case analysis will take a NHS and Personal Social Service perspective, following best UK practice [12]. The base case analysis will use the intention-to-treat (“as-randomised”) dataset. An incremental cost-effectiveness ratio (ICER) will be calculated as the difference in mean costs divided by the difference in numbers of surgical site infections between the interventions. To facilitate clear reporting to different audiences, we will conduct two analyses. CEA1 will comprise a complete case analysis taking a 7–10-day time horizon and focusing on the additional cost of the intervention itself and the cost of subsequent healthcare consultations. Analysis CEA2 will include multiple imputation of missing data and all NHS/Personal Social Service resource use collected in the trial and will take a 4–12-month time horizon for costs, with a sensitivity analysis at 7–10 days.

##### Surgical site infection by 4 months

The presence of a surgical site infection up to 4–12-month period post-randomisation will be assessed. In addition to the clinical assessment at 7 days, the patient’s parent/guardian will be asked if the patient experienced any problems post-surgery. This will then be referred back to sites, where appropriate, to obtain confirmation from clinical notes and if necessary general practitioner notes. This will capture any surgical site infections which occur after the usual expected timeframe in which infections would normally present.

##### Participant/parent satisfaction with nail healing

A scale (3-point Likert scale for children) will be used to measure patient-reported satisfaction with the healing of the nail at 4–12 months post-randomisation. If the child cannot complete this score, a Visual Analogue Scale (VAS) in the form of a measured line with a continuous scale (from 0 to 100) anchored by two verbal descriptors for each extreme symptom will be completed by a parent/guardian and used as a patient proxy for measuring satisfaction with nail healing.

### Sample size

Sample size calculations are based on the co-primary outcomes of surgical site infection and cosmetic appearance at a minimum of 4 months, measured via the Oxford Finger Nail Appearance Score, the development of which was informed by the Zook nail classification scale [8]—a 0–5 ordinal summary score reflecting optimal or suboptimal appearance across the five classification domains. Pilot data from our NINJA-P trial [5] showed a substantial proportion of participants did not have nails with optimal appearance (approximately 35% had two or more suboptimal aspects of appearance, i.e. score of three or less). Based upon a clinically relevant difference of 15% more achieving the optimal appearance score of 5 (from 35 to 50% with a corresponding shift in the other score values) and using a two-sided significance level of 0.05, 332 (166 per group) are required to obtain 90% power based upon a Mann-Whitney *U* test. After allowance for 20% missing data, a total of 416 participants (208 in each group) are required. This calculation was carried out using an extended version of the Excel spreadsheet provided by Walters [13] to allow for a 6-point ordinal outcome. Based upon a lower overall level and a smaller difference in the proportion with a surgical site infection than the one observed in the Miranda et al. [4] observational study (8% vs 1%), this sample size is also sufficient for 90% power at the two-sided 5% significance level. This latter calculation was carried out in Stata 14 using the power twoprop command. The sample size was not adjusted for the use of co-primary outcomes as each outcome is seen as clinically important in its own right and will be evaluated independently.

### Statistical analysis

#### General analysis principles

Principal statistical analyses will be on an “as randomised” (intention-to-treat) basis retaining participants in their randomised allocation groups irrespective of compliance to the allocation or the presence/extent of the nail bed injury. A two-sided 5% significance level will be adopted with associated 95% confidence intervals (CIs) whenever possible using appropriate summary measures (e.g. number of events and percentage for binary measures). The principal analyses will also be carried out on a complete case basis, with sensitivity to missing data explored for the primary outcomes.

Only a single main analysis is planned once the study data collection is complete. No formal stopping rules are accounted for in the sample size, and accordingly, no formal interim analyses are planned. Correspondingly, no adjustment for multiplicity will be undertaken. In the statistical analyses, the two primary outcomes are viewed as clinically important in their own right and will be analysed independently.

In the case where the two co-primary outcomes produce conflicting results (e.g. one outcome favours replacement of the nail, while the other favours discarding), careful interpretation will be required along with consideration of the impact for patients and the healthcare system.

#### Descriptive analyses

**Fig. 1:**
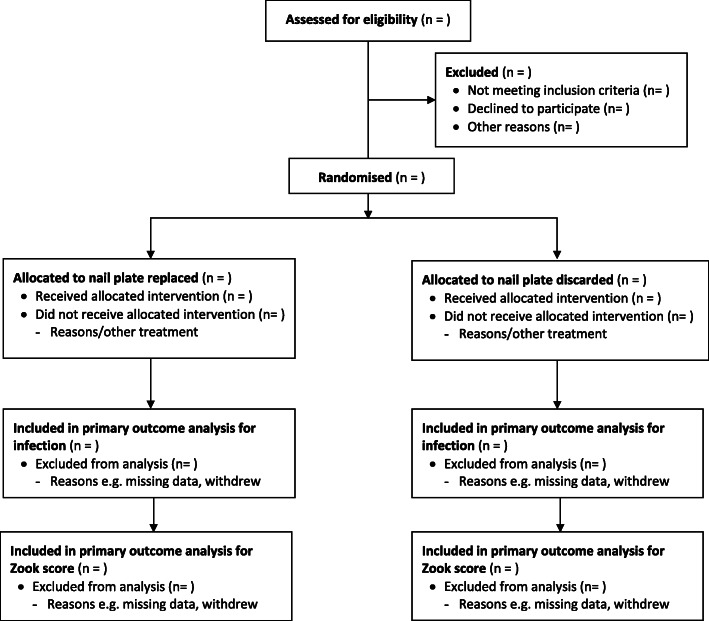
CONSORT flow diagram showing participant flow through trial

The flow of participants through the NINJA trial will be summarised through a Consolidated Standards of Reporting Trials (CONSORT) flow chart [14], which will include the numbers assessed for eligibility, randomised to each trial arm, receiving the allocated treatment, and included in the primary analysis for each of the co-primary outcomes (Fig. 1). Reasons for ineligibility, loss to follow-up, and exclusion from primary analysis will also be detailed, along with the number of patients who withdrew from before each data collection time point.

Baseline characteristics for the participants will be reported both by trial arm and overall, presented as numbers and percentages for categorical variables and as means and standard deviations for continuous variables. No tests of statistical significance for differences between the treatment arms will be carried out for baseline characteristics. The baseline characteristics to be reported will include participant age, gender, and study site at which the participant was randomised (as the sole stratification factor).

#### Withdrawals from treatment and/or follow-up

Numbers and percentages of withdrawals/loss to follow-up together with reasons will be reported by trial arm and compared for each of the time points where outcome data is collected (7–10-day clinical appointment, 4–12-month postal/electronic follow-up). Differential losses between the groups will be informally compared and reasons for any difference explored.

#### Missing data

Patterns of availability for primary and key secondary outcomes from baseline to the end of the follow-up period will be summarised for the two trial arms as the number and percentage of individuals missing for each outcome. Reasons for missingness will be presented if known.

The surgical site infection co-primary outcome is anticipated to have low levels of missing data. There is a system in place to minimise the likelihood of missing surgical site infection data (as described in the “Primary outcomes” section).

A substantial amount of missing outcome data for the Oxford Finger Nail Appearance Score is anticipated. In the case of a high level of missing outcome data for the Oxford Finger Nail Appearance Score, baseline characteristics will be tabulated separately for those with missing outcome data and those with outcome data present.

No imputation is planned for the primary analysis of the co-primary and secondary clinical outcomes or CEA1. CEA2 will use multiple imputation analysis to impute missing data on costs and infections (and health utilities if cost-utility analysis (CUA) can be performed). Chained regression equations will be used to predict missing data based on the observed responses of participants and create sets of multiple datasets containing possible values for missing observations. Subsequent analyses will be performed on the imputed datasets, and results will be combined using Rubin’s rule [15].

#### Compliance

As both interventions are surgical in nature, patient compliance is not relevant in this setting. However, a record will be made of compliance to the surgical procedure. If a patient does not receive their randomised intervention, this will be recorded along with the reason why. This information will be summarised by trial arm.

#### Analysis of primary outcomes

Numbers and percentages of early surgical site infections will be calculated and presented by each of the two treatment groups, nail plate replaced, or nail plate discarded. Rates of surgical site infection in the intervention groups will be compared using logistic regression, adjusting for site as the only stratification factor using the cluster robust option in Stata. The results of the logistic regression will be reported as odds ratios (ORs) together with the associated 95% confidence intervals and *p* values for comparison between the two intervention groups. The unadjusted OR will be reported with the corresponding 95% confidence interval. This analysis will be conducted for the “as randomised” population. In the case of a very low observed infection rate precluding logistic regression, a crude (that is unadjusted for any factors including site) risk ratio will instead be calculated together with the associated 95% confidence intervals and *p* value.

The Oxford Nail Score will be reported both in terms of its individual components and total scores. Numbers and percentages for component scores and the proportion of patients achieving each total score value will be calculated and presented by treatment arm and overall.

As photographs will be assessed by five independent reviewers, the median of the reviewers’ scores will be taken as the score for each photo. From these scores, the median total score will be calculated and reported, as well as a binomial exact 95% confidence interval [16] calculated using the centile command in Stata. The difference in distributions of the Oxford Finger Nail Appearance Scores between intervention groups will be formally assessed by a Mann-Whitney *U* test, and the *p* value of the test will be reported. Ordinal logistic regression with adjustment for site using the cluster robust option in Stata will be used as a secondary method of analysis to assess the difference in scores across the ordinal scale under the assumption of proportional odds between scores, with adjusted and unadjusted ORs reported.

Sensitivity to missing data for the primary outcome of surgical site infection will be assessed using the rctmiss command in Stata [17]. Rctmiss can be used to assess departures from the assumption that outcome data is missing at random. Sensitivity to differential outcome between observed and non-observed data between groups will be investigated over a range of assumptions. Specifically, a pattern-mixture model will be used extending the logistic regression for surgical site infection.

Sensitivity to missing data from the Oxford Finger Nail Appearance Score will be assessed assuming that the values of the missing data are the same in each arm. Two scenarios will be considered:

- All missing score values will be imputed as a 5 (the most optimal score) in both treatment arms.
- All missing score values will be imputed as a 0 (the least optimal score) in both treatment arms.

Each of these scenarios will be analysed using the primary analysis strategy of a Mann-Whitney *U* test.

These sensitivity analyses will be carried out on the “as randomised” population.

#### Analysis of secondary outcomes

Secondary outcomes that will be undertaken as part of the statistical analysis are the pain at dressing change, incidence of surgical site infection by 4 months, and satisfaction with the nail appearance.

Secondary outcomes will be analysed using generalised linear models where possible. Generalised linear models are useful in that they can be utilised to analyse varying types of data which do not necessarily follow a normal distribution. Pain at dressing change will be categorised into either pain or no pain (“Hurts a little bit” and “Hurts a lot” classed as pain, and “Does not hurt” classed as no pain) and will be analysed using logistic regression. Surgical site infection at 4 months will be analysed with logistic regression as per the surgical site infection primary outcome. Parent-reported satisfaction with the nail appearance will be assessed using linear regression, with child-reported satisfaction being analysed using ordinal logistic regression separately. The EQ-5D-Y quality of life outcome will be assessed using linear regression, adjusting for the baseline EQ-5D-Y values recorded pre-surgery. These models will include intervention group as the main independent variable, additionally adjusting for site as the sole stratification variable. Adjusted and unadjusted effect measures will be reported together with 95% confidence intervals and *p* values. Analysis of the secondary outcomes will be carried out on the “as randomised” population on a complete case basis.

#### Safety

The following is a list of events that are deemed “expected” in relation to nail bed injury repairs in this patient population:

- Infection, e.g. local wound infection, felon, osteomyelitis, septic arthritis of distal interphalangeal joint
- Bleeding complications, e.g. over-granulation
- Delayed healing
- Allergic reactions to dressings
- Allergic reaction to local or general anaesthetic
- Airway problems/laryngospasm
- Abnormal nail growth, e.g. split nail, onycholysis
- Synechia
- Altered sensation
- Complex Regional Pain Syndrome, e.g. persistent pain, persistent painful hypersensitivity
- Stiffness

Incidence of these will be captured on participant questionnaires at the follow-up time points at 7–10 days and 4–12 months. If they are deemed serious (as per criteria above), they will be recorded as serious adverse events as soon as the event becomes known to the site trial team or the central trial office.

Frequency and severity of these events will be assessed.

#### Subgroup analysis

There is one pre-specified subgroup analysis of pre-operative antibiotic usage. A treatment-by-subgroup interaction will be used to extend the aforementioned regression models for the co-primary outcomes.

### Health economic analysis

#### Resource use

Resource use for the nail repair surgery will be recorded by the research team in the case report form (CRF) while data for the economic evaluation will be collected during the trial from information gathered via the trial questionnaires given to participants at 7–10 days and 4–12 months after randomisation into the trial. The questionnaires will capture information such as frequency of use of inpatient care, outpatient care, community care, and social care services “because of their fingernail injury”. It will also record direct medical costs (e.g. medications), direct nonmedical costs (e.g. help with housework/childcare and travel), and indirect costs (i.e. carer absenteeism) attributable to the patient’s injury.

#### Health outcomes

An EQ-5D-Y value set in the UK is currently not available [18]. However, if it becomes available by the time of analysis, quality-adjusted life years (QALYs) will be calculated as the area under the baseline-adjusted utility curve of EQ-5D utility scores from baseline and 4-month data using the trapezoidal rule [19].

#### Costing

Unit costs for each resource item associated with the trial will be sourced from the latest national sources such as the NHS Supply Chain Catalogue [20] and the NHS Reference Cost [21]. The base currency of all costs will be the year in which the costing analysis was performed and in UK pounds.

Intra-operative resource use between the treatment arms is expected to be the same except for the extra suture needed to secure the nail plate. The unit cost of suture will be sourced from the latest NHS Supply Chain Catalogue and will be based on the type of suture recorded on the operation form. Since both types of surgery are day cases, the cost of hospitalisation will not be collected as the HRG code is expected to be the same. Unit costs for labour will also not be collected as the number and type of staff (surgeons, anaesthetists, technicians/operating department personnel, nurses, and radiographers) as well as the length of operation are expected to be almost the same in both trial arms.

Unit cost of direct medical costs that are not collected in the trial, such as inpatient care, outpatient care, and community care, will be sourced from the latest available NHS Reference Cost and Personal Social Service Research Unit [22] (Table 1). The unit cost of medication related to nail injury will be sourced using the latest available British National Formulary (BNF) [24] and NHS Electronic Drug Tariff [25]. The base case analysis (taking an NHS perspective) will exclude medications that are recorded on the questionnaire as having been bought without prescription. All medications are assumed to be in oral solution (regardless of the patient’s age) unless stated otherwise. The medication cost taken over the study period will be computed using the cost per dose for each product and the mean quantity taken per day during the reported number of days. If less than one bottle will be used to treat the nail bed injury, the costing analysis will include the entire cost of the whole bottle prescribed. As the participant’s parent/guardian is not required to indicate the dose of the medication, the defined daily dose for each medication will be taken from the BNF website after consulting expert opinion. Cost of healthcare resource use per patient will be computed by multiplying the frequency of health resource utilisation rate reported by the parent/guardian with the unit cost of each resource item.

**Table 1:** Unit costs of health and social care items and additional financial cost items due to nail injury

Resource item	Unit	Source
**NINJA cost**		
6/0 interrupted vicryl rapide suture	Each	NHS Supply Chain Catalogue [20]
7/0 interrupted vicryl rapide suture	Each	NHS Supply Chain Catalogue [20]
**Other direct medical cost**		
Inpatient care (due to complications of nail injury)	Visit	NHS Reference Cost [21]
Outpatient care		
Plastic or hospital dressing clinic	Visit	NHS Reference Cost [21]
Pathology (blood tests or swabs)	Test	NHS Reference Cost [21]
Radiology (x-rays)	Visit	NHS Reference Cost [21]
Physiotherapy	Visit	NHS Reference Cost [21]
Emergency department (due to nail injury)	Visit	NHS Reference Cost [21]
Community care		
General practitioner (surgery)	Visit	PSSRU [22]
General practitioner (home)	Visit	PSSRU [22]
General practitioner	Call	PSSRU [22]
Practice nurse (surgery)	Visit	PSSRU [22]
Calls to NHS 111 (formally NHS Direct)	Call	Financial Times, 2017 [23]
Medication	Each	BNF [24], NHS Electronic Drug Tariff [25]
**Direct nonmedical cost**		
Missed work (parent/guardian)	–	Trial
Travel (parent/guardian)	–	Trial
Child care (parent/guardian)	–	Trial
**Indirect cost**		
Median wage	Day	Office for National Statistics [26]

*BNF* British National Formulary, *PSSRU* Personal Social Service Research Unit

CEA1 will include the cost of suture and consultations with healthcare professionals that occur within 7–10 days. CEA2 will include all NHS and Personal Social Service resources captured on the questionnaires in the base case analysis, and a sensitivity analysis will be done taking a societal perspective. Unit costs for direct nonmedical cost items, such as help with housework/childcare and travel incurred by participant’s carer, will be obtained directly from the questionnaires. Cost of absenteeism will be computed using the human capital approach where the daily median wage will be multiplied by the number of days the participant’s carer had to take time off work due to participant’s nail injury. The daily median wage will be obtained from the Office for National Statistics [26].

#### Data analysis: CEA1

Data on the total cost per patient over the first 7–10 days and the presence/absence of infections will be bootstrapped (with 1000 replications) to estimate the mean and 95% CI for the incremental cost, the number of infections avoided, and the cost per infection avoided. Running 1000 bootstraps has previously been shown to give stable results [27]. We will plot the cumulative mean difference in cost and infection rate and their 95% CI against the number of bootstraps to verify visually whether the number of bootstraps is sufficient to give stable results. To simplify reporting and interpretation of results, patients with missing data will be excluded from the analysis and there will be no stratification by study site or adjustment for covariates. No discounting will be applied due to the short time horizon.

#### Data analysis: CEA2

Resource use items will be summarised by trial allocation group, and follow-up period and differences between groups will be analysed using *t* tests for continuous variables and Pearson’s chi-squared (*χ*^2^) test for categorical variables. Means and standard deviations for values of each cost category will be estimated by treatment allocation and follow-up period. Differences in mean costs in each category will be assessed using *t* tests.

The CEA will be conducted by bootstrapping *M* multiple imputations of data on total costs and numbers of infections, where *M* equals the percentage of patients with missing data on any variable included in the cost-effectiveness analysis. We will do 100 bootstrap replications for each of the *M* imputed datasets and combine the results using Rubin’s rule; convergence will be checked in the same way as for CEA1. The proportion of bootstraps in which nail replacement is cost-effective will be plotted against the willingness-to-pay to avoid an infection. There will be no discounting of costs or outcomes because cost-effectiveness will be determined within a year.

In addition to the CEA, a CUA will be conducted as a secondary analysis if a validated EQ-5D-Y value set is available at the time of analysis and an ICER will be computed as the difference in mean costs divided by the differences in mean QALYs between the interventions, with 95% CI estimated using bootstrapping and Rubin’s rule. The National Institute for Health and Care Excellence [28] cost-effectiveness threshold of £20,000 to £30,000 per additional QALY will be used to determine the cost-effectiveness of replacing versus discarding the nail plate after nail bed repair surgery. An intervention with an ICER below the £20,000 per QALY threshold will be considered cost-effective.

The net monetary benefit of replacing versus discarding nail plate will be computed across different cost-effectiveness thresholds. Cost-effectiveness acceptability curves will be drawn to summarise the uncertainty around the results of the economic evaluation. One-way sensitivity analysis will be performed to explore the effects of extending the study perspective (i.e. societal perspective), taking a 7–10-day time horizon, and assessing the impact of missing data on the ICERs (i.e. using complete case analysis).

Findings of this economic evaluation will be reported in accordance with the Consolidated Health Economic Evaluation Reporting Standards (CHEERS) statement for the reporting of health economic evaluations [29].

#### Statistical packages

All analysis will be carried out using Stata [30] or R [31] statistical software. The relevant package and version number used for analysis will be recorded and reported.

## Discussion

The NINJA trial will provide data on the clinical effects of replacing the nail plate compared to discarding the nail plate following the repair of a nail bed injury.

This paper gives details of the planned statistical and health economic analyses for the NINJA trial, with the aim of reducing the risks of reporting bias and data-driven results [32]. Any changes or deviations from the analysis outlined in this paper will be described and justified fully in the final report.

### Trial status

Recruitment to the NINJA trial closed on 9 July 2019. A total of 451 participants were recruited from 20 sites around the UK. Follow-up for the trial outcome data will be completed in 2020. Statistical and economic analyses of all trial outcomes will be conducted thereafter.

## Data Availability

Not applicable.

## Abbreviations

ATC: Anatomical Therapeutic Chemical
BAPRAS: British Association of Plastic, Reconstructive and Aesthetic Surgeons
BSSH: British Society for Surgery of the Hand
CAT: Confirmation of additional treatment
CEA: Cost-effectiveness analysis
CHEERS: Consolidated Health Economic Evaluation Reporting Standards
CONSORT: Consolidated Standards of Reporting Trials
CRF: Case report form
CUA: Cost-utility analysis
EQ-5D-Y: EuroQoL 5-Dimension Youth
HRQoL: Health-related quality of life
ICER: Incremental cost-effectiveness ratio
NHS: National Health Service
NINJA: Nail bed INJury Analysis
OR: Odds ratio
PedsQL: Paediatric Quality of Life Inventory
QALY: Quality-adjusted life year
SHEAP: Statistical and health economic analysis plan
VAS: Visual Analogue Scale
